# Characterization of an Aldehyde Oxidoreductase From the Mesophilic Bacterium *Aromatoleum aromaticum* EbN1, a Member of a New Subfamily of Tungsten-Containing Enzymes

**DOI:** 10.3389/fmicb.2019.00071

**Published:** 2019-01-31

**Authors:** Fabian Arndt, Georg Schmitt, Agnieszka Winiarska, Martin Saft, Andreas Seubert, Jörg Kahnt, Johann Heider

**Affiliations:** ^1^Faculty of Biology, Philipps-Universität Marburg, Marburg, Germany; ^2^Jerzy Haber Institute of Catalysis and Surface Chemistry, Polish Academy of Sciences, Kraków, Poland; ^3^Faculty of Chemistry, Philipps-Universität Marburg, Marburg, Germany; ^4^Max-Planck-Institut für Terrestrische Mikrobiologie, Marburg, Germany; ^5^LOEWE Center for Synthetic Microbiology, Philipps-Universität Marburg, Marburg, Germany

**Keywords:** aldehyde oxidoreductase, tungsten cofactor, Fe-S cluster, flavin, enzyme kinetics

## Abstract

The biochemical properties of a new tungsten-containing aldehyde oxidoreductase from the mesophilic betaproteobacterium *Aromatoleum aromaticum* EbN1 (AOR*_Aa_*) are presented in this study. The enzyme was purified from phenylalanine-grown cells of an overexpressing mutant lacking the gene for an aldehyde dehydrogenase normally involved in anaerobic phenylalanine degradation. AOR*_Aa_* catalyzes the oxidation of a broad variety of aldehydes to the respective acids with either viologen dyes or NAD^+^ as electron acceptors. In contrast to previously known AORs, AOR*_Aa_* is a heterohexameric protein consisting of three different subunits, a large subunit containing the W-cofactor and an Fe-S cluster, a small subunit containing four Fe-S clusters, and a medium subunit containing an FAD cofactor. The presence of the expected cofactors have been confirmed by elemental analysis and spectrophotometric methods. AOR*_Aa_* has a pH optimum of 8.0, a temperature optimum of 40°C and is completely inactive at 50°C. Compared to archaeal AORs, AOR*_Aa_* is remarkably resistant against exposure to air, exhibiting a half-life time of 1 h as purified enzyme and being completely unaffected in cell extracts. Kinetic parameters of AOR*_Aa_* have been obtained for the oxidation of one aliphatic and two aromatic aldehydes, resulting in about twofold higher *k*_cat_ values with benzyl viologen than with NAD^+^ as electron acceptor. Finally, we obtained evidence that AOR*_Aa_* is also catalyzing the reverse reaction, reduction of benzoate to benzaldehyde, albeit at very low rates and under conditions strongly favoring acid reduction, e.g., low pH and using Ti(III) citrate as electron donor of very low redox potential. AOR*_Aa_* appears to be a prototype of a new subfamily of bacterial AOR-like tungsten-enzymes, which differ from the previously known archaeal AORs mostly by their multi-subunit composition, their low sensitivity against oxygen, and the ability to use NAD^+^ as electron acceptor.

## Introduction

**FIGURE 1:**
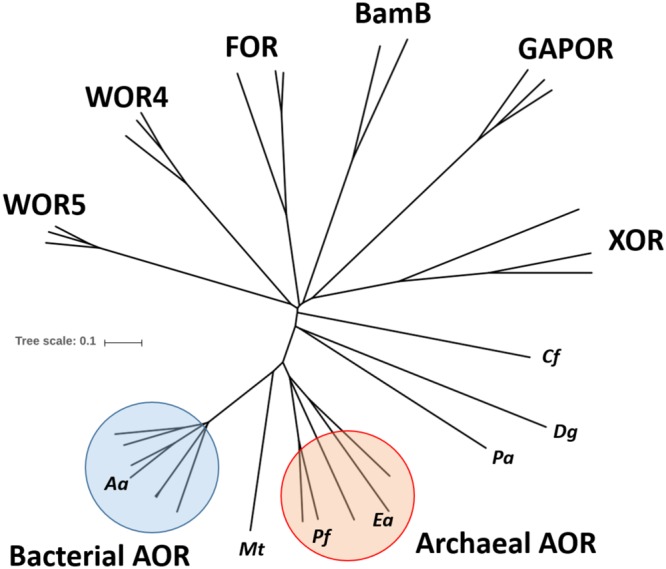
Phylogenetic tree of the AOR enzyme family. The amino acid sequences of the large subunits of selected members of the AOR family were aligned and used to construct an unrooted phylogenetic tree. The identities of the major subfamilies is indicated and some individual sequences are labeled by abbreviated species names: *Aa, Aromatoleum aromaticum* EbN1; *Mt, Moorella thermoacetica*; *Pf, Pyrococcus furiosus*; *Ea, Eubacterium acidaminophilum*; *Pa, Pyrobaculum aerophilum*; *Dg, Desulfovibrio gigas*; *Cf, Clostridium formicoaceticum*. *Pa, Dg*, and *Cf* represent sequences of biochemically characterized homodimeric AORs which do not coincide with either the bacterial or archaeal AOR subfamily. Accession numbers can be found in the supplement (Supplementary Table S1).

Many bacteria and archaea use either molybdenum (Mo) or tungsten (W) as catalytic transition metals in enzymes catalyzing key steps of metabolism, many of which are of fundamental importance for global nutrient cycles (Hille, 2002; Hille et al., 2014). Molybdo- and tungstoenzymes contain the metal bound by an organic molybdopterin (MPT) cofactor and combine a variety of metabolic capabilities, particularly acting as dehydrogenases, oxidases, hydroxylases, hydratases or reductases (Kletzin and Adams, 1996; Hille, 2002; Hille et al., 2014; Maia et al., 2015). Many molybdo- or tungstoenzymes also contain Fe-S clusters and various other redox active cofactors (Hille et al., 2014; Maia et al., 2015). Based on sequence similarities of their catalytic subunits and similar cofactor compositions, these enzymes are grouped into four distinct enzyme families, the sulfite oxidase, xanthine dehydrogenase, DMSO reductase, and aldehyde oxidoreductase (AOR) families (Kletzin and Adams, 1996; Hille, 2002; Hille et al., 2014; Maia et al., 2015). Among these, W-dependent enzymes are known in the DMSO-reductase family, which contains mostly molybdoenzymes, and in the AOR family, which consists almost exclusively of tungstoenzymes. In both families, the metal is ligated to two MPT-derived cofactors which provide four sulfur ligands. The W-dependent enzymes of the DMSO reductase family contain two molybdopterin guanine dinucleotides (MGD) as ligands per W and are paralogous to Mo-containing isoenzymes, such as formate dehydrogenases, formyl-methanofuran dehydrogenases or nitrate reductases (Maia et al., 2015), whereas the enzymes of the AOR family contain two MPT per W (Chan et al., 1995; Hu et al., 1999). The only AOR-type enzyme described so far as a Mo-enzyme is a poorly characterized α-hydroxyacid oxidoreductase from *Proteus vulgaris* (Trautwein et al., 1994). Most W-dependent enzymes of the AOR family described to date are from hyperthermophilic archaea (Kletzin and Adams, 1996). For example, five hyperthermophilic and extremely O_2_-sensitive W-enzymes of the AOR family are encoded in the genome of *Pyrococcus furiosus*, which have all been described as aldehyde oxidoreductases of various specificities (Kletzin and Adams, 1996; Roy and Adams, 2002; Bevers et al., 2005) and represent five separate subfamilies in a phylogenetic tree of the AOR family enzymes (Figure 1). The most important of these are the homodimeric aldehyde oxidoreductases (AOR *sensu stricto*) (Mukund and Adams, 1991), the homotetrameric formaldehyde oxidoreductases (FOR) (Mukund and Adams, 1993), whose structures have been solved (Chan et al., 1995; Hu et al., 1999), and the monomeric glyceraldehyde-3-phosphate oxidoreductases (GAPOR) (Mukund and Adams, 1995). Finally, two further tungsten-containing oxidoreductases, WOR-4 (Roy and Adams, 2002) and WOR-5 (Bevers et al., 2005) have been purified, but their physiological relevance is unknown. Further orthologs of these enzymes have also been characterized from various other hyperthermophilic archaeal species, such as *Thermococcus litoralis* (Kletzin et al., 1995), *T. paralvinellae* (Heider et al., 1995), *Methanobacterium thermoautotrophicum* (Bertram et al., 1994) or *Pyrobaculum aerophilum* (Hagedoorn et al., 2005). In addition, they also have been described in some bacteria as “carboxylic acid reductase” (CAR, e.g., in *Moorella thermoacetica*; White et al., 1989; Strobl et al., 1992; Huber et al., 1995), or as AORs from *Clostridium formicoaceticum* (White et al., 1991), *Eubacterium acidaminophilum* (Rauh et al., 2004) or *Desulfovibrio gigas* (Hensgens et al., 1995). Recently, an enzyme of the AOR family from the anaerobic thermophilic bacterial genus *Caldicellulosiruptor* has been identified as a member of a new subclass called XOR (Scott et al., 2015). Finally, a separate branch of W-dependent enzymes of the AOR family was recently discovered in obligatory anaerobic aromatic-degrading bacteria, which were identified as benzoyl-CoA reductases (Kung et al., 2009). These enzymes are very large multi-subunit complexes and contain an AOR-type subunit with a modified W-*bis*-MPT cofactor, which exhibits an additional unknown small ligand to the W atom (Weinert et al., 2015).

Recently, a tungsten-dependent AOR-like enzyme was detected in the denitrifying betaproteobacterium *Aromatoleum aromaticum* EbN1 (Debnar-Daumler et al., 2014). The enzyme is induced during anaerobic growth on phenylalanine (Phe) and a number of other substrates, although the degradation of aldehydes in the respective pathways occurs mainly via NAD(P)^+^-dependent dehydrogenases (Schmitt et al., 2017). Moreover, the amounts of this AOR in different cell batches are usually rather low and the specific activities vary considerably between batches. Therefore, its physiological function is assumed to be the degradation of aldehyde intermediates (e.g., phenylacetaldehyde during Phe metabolism) to avoid accumulation of toxic concentrations. To establish a more reliable source of AOR, a deletion mutant of *A. aromaticum* EbN1 was constructed which lacked the gene for the specific phenylacetaldehyde dehydrogenase (*pdh*) usually employed in anaerobic Phe metabolism. This strain indeed consistently produced AOR in high amounts and specific activities (Schmitt et al., 2017). In this report, we present the purification of *A. aromaticum* EbN1 AOR (henceforth called AOR*_Aa_*) from this deletion strain and report its biochemical properties and deviant features from previously known AOR isoenzymes.

## Materials and Methods

### Growth of Bacteria

*Aromatoleum aromaticum* EbN1 strain SR7Δ*pdh* (Schmitt et al., 2017) was grown anaerobically in ascorbate-free minimal medium using phenylalanine as sole carbon source and nitrate as electron acceptor, as described previously (Rabus and Widdel, 1995). Phenylalanine and nitrate were supplied at concentrations of 1 and 3.5 mM, respectively, and discontinuously re-fed at the same concentrations when nitrate was consumed. Cultures were incubated at 28°C in stoppered 1 liter flasks or in a 200 L fermenter. Growth was followed by determining the increase in optical density at 578 nm and the consumption of nitrate. The standard culture medium for *A. aromaticum* EbN1 contained 150 nM Na_2_MoO_4_ and 23 nM Na_2_WO_4_ and was prepared with deionized water.

### Preparation of Cell Extracts

Cells were harvested by centrifugation at 17,000 × *g* and 4°C for 20 min. Sedimented cells were immediately frozen and stored at -80°C. All further steps were performed under anoxic conditions. For preparation of extracts, cells were suspended in one volume of 100 mM Tris-HCl buffer (pH 8.0) with 10% glycerol or for subsequent chromatographic separation in 20 mM Bis-Tris buffer (pH 6.2) containing 0.05 mg DNase I per ml and 10% glycerol. Cell suspensions were disrupted by sonication or passed thrice through a French pressure cell press. Cell debris and membranes were removed by ultracentrifugation at 100,000 × *g* and 4°C for 1 h. The supernatants were stored anaerobically with 10% (vol/vol) glycerol at -80°C until use.

### Purification of Aldehyde Oxidoreductase

For purification of AOR*_Aa_* a three-step strategy was applied under anoxic conditions, using thoroughly degassed buffers at 16°C while collecting fractions at 4°C. Cell extracts were passed through a 0.45 μm filter before application to a column. First, cell-free extracts were loaded to a DEAE-sepharose Fast Flow column (26/12) equilibrated with buffer A (20 mM Bis-Tris buffer, pH 6.2) and AOR was eluted by a step gradient with added buffer B (20 mM Bis-Tris, pH 6.2, 1 M NaCl) to yield 400 mM NaCl. Active fractions were pooled and re-buffered to buffer C (5 mM MES, pH 6.8, 1 mM CaCl_2_) using a HiPrep (26/10) desalting column (GE-Healthcare). In a second step, the fractions were applied to a ceramic hydroxyapatite column (CHT-I, 70 ml) equilibrated with buffer C. Fractions with AOR*_Aa_* activity eluted early in a linear gradient between buffers C and D (5 mM MES, pH 6.8, 400 mM potassium phosphate pH 6.8) when 15 mM phosphate was applied. The pooled fractions of AOR*_Aa_* were concentrated under anoxic conditions by ultrafiltration (Amicon) using a 30 kDa cutoff. Finally, gel filtration on Superdex 200 was performed using a 120 ml column (16/60) equilibrated with buffer E (100 mM Tris-HCl, pH 8.0, 150 mM NaCl). Activity assays or ultrafiltration was performed anaerobically. Each buffer were supplemented with 10% glycerol (v/v) and AOR fractions were stored anaerobically at -80°C until further use without significant loss of AOR activity.

### Enzyme Activity Assays

Enzyme activity were assayed photometrically at 28°C. Activity of AOR*_Aa_* was assayed under anoxic conditions as described previously (Schmitt et al., 2017), but using 100 mM Tris-HCl (pH 8.0) as standard buffer. The reactions were started by the addition of different aldehydes (1 mM) and followed at 600 nm to record reduction of benzylviologen (𝜀 = 7400 M^-1^ cm^-1^), and at 340 nm for reduction of NAD^+^ to NADH (𝜀 = 3400 M^-1^ cm^-1^), respectively. The reverse reaction was assayed as reduction of benzoate to benzaldehyde in 50 mM MES/KOH buffer (pH 5.0), containing 1.2 mM Ti(III) citrate, 20–100 mM benzoate and 10–20 μg of protein. This was incubated at 20°C for 15 min, then 3 mM 3-nitrophenylhydrazine were added to the reaction mixture to trap the aldehydes formed, and the assays were incubated for additional 2–3 h. The analysis of the products was performed by TLC using Silica gel 60 F_254_ material (Merck). The TLC plates were loaded with 15–20 μl of the reaction mixtures or appropriate controls and developed using a mobile phase of toluene/acetone/isopropanol/acetic acid (17:3:3:1).

### Flavin Cofactor Analysis

Flavin cofactor contents were determined as described in Leutwein and Heider (2002). Cofactor of purified AOR was released by denaturation for 10 min at 95°C and subsequent centrifugation (17,000 × *g*, 10 min). The supernatant was used for UV-Vis- and fluorescence spectroscopy. Phosphodiesterase (80 mU/ml) was added to the supernatant to observe changes in flavin fluorescence. The flavin content was estimated from UV-Vis spectrum by the molar extinction coefficient of FAD at 450 nm (𝜀 = 11.3 mM^-1^ cm^-1^), fluorescence intensity was determined by excitation at 450 nm and emission at 524 nm. For quantification a freshly prepared FAD solution was used.

### Phylogenetic Analysis

The amino acid sequences of tungsten containing oxidoreductases were analyzed by BLAST searches against the NCBI database using default settings. Alignment of selected sequences was performed by Clustal Omega^1^ (Sievers et al., 2011), and phylogenetic trees were constructed by iTol^2^ (Letunic and Bork, 2007).

### Other Methods

Protein concentration was determined by the method of Bradford using bovine serum albumin as standard (Coligan et al., 2004). Proteins were separated by discontinuous SDS-PAGE. Molecular masses of proteins were estimated by gel filtration (Superdex 200) and Ferguson plots, using non-denaturing gel electrophoresis with polyacrylamide concentrations of 6, 7, 8, and 10% (Coligan et al., 2004). Standards were bovine serum albumin and its oligomers (67–268 kDa) and ovalbumin (45 kDa). Metal contents of protein fractions with enriched AOR activities and of respective controls were analyzed by inductively coupled plasma mass spectrometry (ICP-MS). The identities of proteins separated by SDS-PAGE were determined from the masses of tryptic fragments, using a 4800 Proteomics Analyzer (MDS Sciex, Concord, ON, Canada). MS data were evaluated against an in-house database using Mascot embedded into GPS explorer software (MDS Sciex, Concord, ON, Canada).

## Results

### Purification of AOR*_Aa_*

We have reported previously that *A. aromaticum* EbN1 produces a W-containing AOR (AOR*_Aa_*) when grown under nitrate-reducing conditions with phenylalanine (Phe), which catalyzes the same reaction of the degradation pathway as a simultaneously induced phenylacetaldehyde dehydrogenase (PDH), oxidation of phenylacetaldehyde to phenylacetate (Debnar-Daumler et al., 2014; Schmitt et al., 2017). Unfortunately, the amount of AOR*_Aa_* present in cell extracts has been found to be unreliable in different cell batches and too low to allow its purification (Debnar-Daumler et al., 2014). Therefore, we constructed a mutant of *A. aromaticum* EbN1 in which the *pdh* gene was deleted and replaced by a gentamicin resistance gene, leaving only AOR*_Aa_* to contribute to phenylacetaldehyde oxidation. When grown with Phe, this mutant indeed reliably contained five-fold higher AOR*_Aa_* activities than observed in wild type cells (Schmitt et al., 2017). The mutant was grown in 30 l-scale on Phe to produce cell mass for the purification of the enzyme. Because the W-content of the first enzyme preparation was lower than expected, the tungstate concentration was increased from 23 nM of the standard medium to 400 nM for the fermenter culture. The measured doubling time of 33 h was similar to that measured in 1-l-cultures, corresponding to a derived consumption rate of 6–11 nmol min^-1^ (mg protein)^-1^. The fermenter culture yielded 108 g wet cell mass per 30 L, which was used to purify AOR*_Aa_*.

**FIGURE 2:**
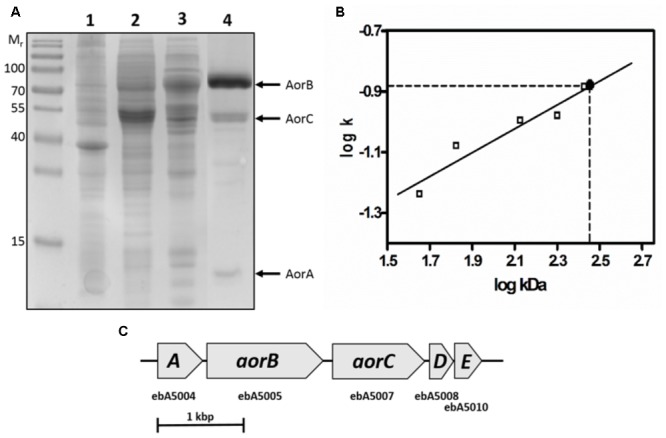
Composition of AOR*_Aa_*. **(A)** SDS-PAGE analysis of the respective active fractions during purification of AOR*_Aa_*. Lanes: Molecular mass standard, cell extract (1), DEAE-sepharose pool (2), hydroxyapatite pool (3), gel filtration pool (4). The corresponding subunits are indicated at the right margin. **(B)** Ferguson plot analysis of native AOR*_Aa_*. The slopes of the relative migration rates vs. concentrations of native polyacrylamide gels (*k*) are plotted against the standard proteins masses in double-logarithmic scale, yielding a mass of ca. 280 kDa for the AOR*_Aa_* complex. **(C)** Operon structure of the genes coding for the subunits of AOR*_Aa_*. The gene products of *aorABC* have been identified as the subunits of the enzyme, whereas *aorD* and *aorE* code for putative maturation factors for the W-cofactor.

Cell extracts were produced by breaking the suspended cells via a French press cell and centrifugation for 1 h at 100,000 × *g*. The extracts contained BV-coupled AOR*_Aa_* activities with a specific activity of 0.278 μmol min^-1^ mg^-1^. AOR*_Aa_* was purified by column chromatography under anoxic conditions. Purification included subsequent chromatographic steps on DEAE-sepharose, hydroxyapatite and gel filtration on Superdex 200. A typical enrichment protocol is given in Table 1, and an SDS-gel analysis of the respective fractions is shown in Figure 2. AOR was enriched about 70-fold with a recovery of 29%, suggesting an abundancy of ca. 1.5% of the total soluble protein content in the cells.

**Table 1:** Purification of AOR*_Aa_*.

Step	Protein [g]	Activity [kU]	Yield [%]	Sp. act. (PAld) [μmol min^-1^ mg^-1^]	Enrichment
Soluble cell extract	10.4	2.9	100	0.28	1.0
DEAE Sepharose	1.40	2.1	82	1.4	5.4
Hydroxyapatite (CHT-I)	0.289	2.0	72	7.0	26
Superdex 200	0.044	0.82	29	19	70

*Specific activities are given in μmol min^-*1*^ mg^-*1*^ (U), using an enzyme assay with phenylacetaldehyde as substrate and BV as electron acceptor.*

### Molecular and Spectroscopic Properties

Surprisingly, purified AOR*_Aa_* consisted of three different subunits of 66, 46, and 17 kDa, which were separated by SDS-PAGE (Figure 2), contrasting to the previously known single subunit AORs present in Archaea (Mukund and Adams, 1991; Heider et al., 1995). Analysis of the respective Coomassie-stained bands from SDS-gels by MALDI-TOF analysis of tryptic fragments confirmed their identity as the gene products of *ebA5004, ebA5005*, and *ebA5007* from the genome of *A. aromaticum* EbN1 (Rabus et al., 2005), which were therefore renamed *aorABC* (Supplementary Table S2). The *aorA* gene codes for the small subunit which is predicted to contain four [Fe_4_S_4_]-clusters, *aorB* codes for the W-cofactor binding large subunit predicted to contain another [Fe_4_S_4_]-cluster, and *aorC* codes for a predicted FAD-containing medium-sized subunit. Moreover, the operon appears to contain two additional genes, *aorD* and *aorE* (Figure 2), which code for proteins resembling the Mo-cofactor maturation factor MoaD. These proteins were not present in the purified enzyme, but may play a role in W-cofactor biosynthesis. The native mass of AOR*_Aa_* was determined by gel filtration and Ferguson plot analysis (Figure 2) as approximately 280 kDa, suggesting an α_2_β_2_γ_2_ composition of the enzyme.

An elemental analysis of purified AOR*_Aa_* by ICP-MS analysis revealed the presence of 1.8 W, 38 Fe, 6.2 P, and 2.3 Mg per α_2_β_2_γ_2_ holoenzyme, whereas no traces of Mo were detected (Table 2). Except for the P-content, these values fit very well with the expected cofactor content based on the structure of archaeal AOR and the predictions from the sequences, namely two W (in AorB), ten [Fe_4_S_4_]-clusters (in AorA and AorB), two Mg and four P as constituents of the W-cofactors in AorB, and four more P in the FAD cofactors of AorC. We only recorded a lower content of P than expected (6.2 instead of 8) which may be explained by a partial occupancy of the AorC subunits with FAD cofactors. This interpretation is supported by the element composition of a preliminary batch of purified AOR*_Aa_* from cells grown with a lower tungstate concentration, which contained only 0.4 W, 4.4 P, and 1.0 Mg, but 50 Fe per α_2_β_2_γ_2_ holoenzyme, suggesting a full [Fe_4_S_4_]-cluster content, but only partial occupancy of both the W- and the FAD-cofactors in this preparation (Table 2). Taking the Mg content of this preparation as indication of the presence of total MPT cofactors (1 Mg corresponds to 2 P), the remaining P content of 2.4 suggests a very similar FAD content in both AOR*_Aa_* preparations.

**Table 2:** Elemental composition of AOR*_Aa_*.

	AOR*_Aa_* batch 1	AOR*_Aa_* batch 2	
Element	[mol/mol AOR]	[mol/mol AOR]	Expected
W	0.37	1.77	2
Mo	n.d.	n.d.	0
Fe	50.9	38.3	40
P	4.37	6.19	8
Mg	1.01	2.32	2

*ICP/MS analysis of two batches of purified AOR from A. aromaticum EbN1. Batch 1 was from cells grown in standard medium (23 nM tungstate), batch 2 was from cells supplied with an elevated tungstate concentration (400 nM). Values are calculated for a predicted size of the AOR_*Aa*_ complex of 260 kDa. The expected values are based on a hypothetical α_*2*_β_*2*_γ_*2*_ composition containing the predicted cofactors. n.d., not detected.*

**FIGURE 3:**
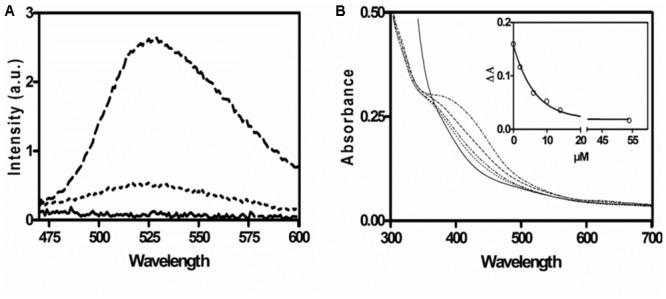
Spectroscopic characterization of AOR*_Aa_*. **(A)** Fluorescence emission spectrum of the supernatant of denatured AOR*_Aa_*. Buffer control (solid line); emission spectrum of supernatant without (dotted line) and with phosphodiesterase treatment (broken line). **(B)** UV-Vis spectra of untreated AOR*_Aa_* (2 μM; upper line) and of the same sample after adding increasing amounts of phenylacetaldehyde (lower lines) and after full reduction with a surplus of dithionite (0.1 mM; solid line). The insert shows the decrease of absorption at 420 nm with the concentration of added substrate.

The presence of FAD in AOR*_Aa_* was confirmed by fluorescence spectroscopy. The supernatant of heat-inactivated enzyme exhibited a typical flavin spectrum with an excitation maximum at 450 nm, and an emission maximum at 528 nm. Subsequent treatment with phosphodiesterase led to a five-fold increased fluorescence at pH 7, indicating the presence of FAD (Figure 3). The flavin content was estimated by comparing the fluorescence at 528 nm after phosphodiesterase treatment with an FMN standard and yielded 0.6 mol flavin per mol α_2_β_2_γ_2_ holoenzyme. This represents an even lower FAD content than inferred by the P content, suggesting that some flavin may have been lost by coprecipitation with the denatured protein.

The UV-Vis spectrum of AOR*_Aa_* is indicative of a protein containing [Fe_4_S_4_]-clusters, exhibiting a broad absorption shoulder at 400–450 nm. The flavin cofactors were not visible in the spectra, probably because the Fe-S-clusters dominated the spectra due to their high extinction coefficients (Roat-Malone, 2003). As expected, the absorbance of the [Fe_4_S_4_]-clusters was quenched upon their reduction, either by the substrate phenylacetaldehyde or by the unspecific reductant dithionite (Figure 3). The recorded absorption value of 0.3 at 390 nm for the preparation of AOR*_Aa_* used in this experiment (2 μM of holoenzyme) is consistent with the expected value for 10 [Fe_4_S_4_]-clusters (assuming a partial extinction coefficient per Fe of 3.8 mM^-1^ cm^-1^; Roat-Malone, 2003). Moreover, stepwise reduction with either phenylacetaldehyde or dithionite indicated full reduction at 15–20 μM reductant (Figure 3), which correlates well with the predicted electron loading capacity of 18 electrons per AOR holoenzyme (two W-cofactors equal four, 10 [Fe_4_S_4_]-clusters equal ten, and two FAD cofactors equal four electrons).

### Dependence of AOR*_Aa_* on pH and Temperature

**FIGURE 4:**
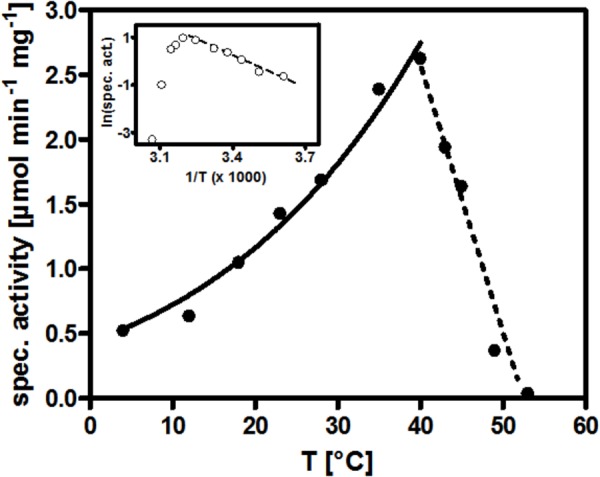
Temperature dependence of AOR*_Aa_*. The curve connecting the specific activity values measured from 4°C up to 40°C represents their mathematical fit against the Arrhenius equation. The insert shows the corresponding Arrhenius plot.

We have recorded the pH dependence of the BV- and NAD^+^-coupled reactions of AOR*_Aa_* separately and obtained optima at pH 8.0 for the oxidation of phenylacetaldehyde with either electron acceptor, using a Tris-HCl buffer system (data not shown). Moreover, we recorded the temperature dependence of AOR, using the BV-coupled oxidation of phenylacetaldehyde. We obtained a relatively high temperature optimum of 40°C, compared to the growth range of the host organism which does not extend to more than 30°C (Rabus and Widdel, 1995). The activity profile of AOR fitted well to the Arrhenius equation between 4°C and the maximum at 40°C, then the activity declined steeply to none at more than 50°C (Figure 4). We calculated an activation energy of 33 kJ/mol from the exponential part of the curve.

**FIGURE 5:**
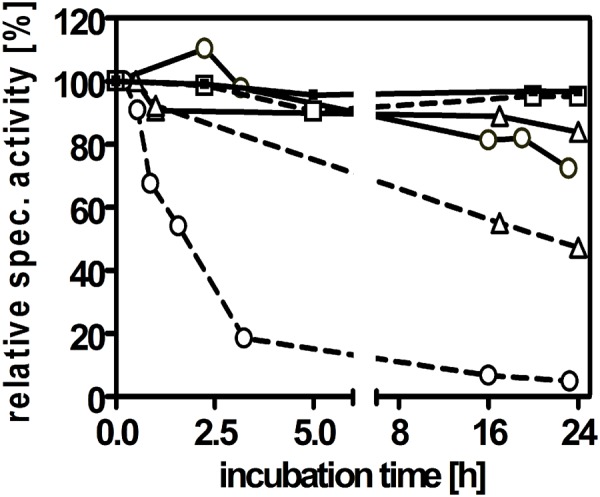
Sensitivity of AOR*_*Aa*_* against air exposure. The enzyme was analyzed for activity after storage at 20°C in the presence or absence of oxygen in three stages during its purification, namely in cell extract (squares), after DEAE-sepharose chromatography (triangles) or pure enzyme after gel filtration (circles). The anaerobically incubated control samples are indicated by solid, those exposed to air by broken connecting lines.

In contrast to archaeal AOR, AOR*_Aa_* appeared to be much more stable when exposed to air, especially in crude cell extracts. We therefore investigated its stability to air exposure at different stages of the purification. We confirmed our initial observation that AOR*_Aa_* is completely unaffected by air in cell extracts and still showed 100% activity after 24 h of exposure, as compared to control experiments with anaerobically incubated enzyme. However, oxygen sensitivity of the enzyme was observed at the later steps during its purification, which apparently increased with the purity of AOR*_Aa_* (Figure 5). Pure AOR*_Aa_* showed a half-life time of 1 h in air and was almost completely inactivated after 3 h, whereas an anaerobically incubated aliquot retained full activity for 24 h (Figure 5). Therefore, AOR*_Aa_* appears to be protected by additional factors against oxygen inactivation in the extract, but the pure enzyme still is remarkably stable in air on its own, compared to the archaeal AOR enzymes, which are inactivated within minutes of exposure (Mukund and Adams, 1991; Heider et al., 1995).

### Catalytic Properties of AOR*_Aa_*

Because of the presence of the additional subunits of AOR*_Aa_* compared to the archaeal enzymes, we presumed that it may accept NAD^+^ or NADP^+^ as additional electron acceptors next to viologen dyes. Indeed, we recorded high activities of aldehyde oxidation with NAD^+^ as electron acceptor using purified AOR*_Aa_*, but none with NADP^+^. The NAD^+^-coupled specific activities were lower than those coupled with BV and ranged at around 45–75% of the latter, dependent on the substrate used (Table 3). AOR*_Aa_* showed a very broad substrate range and oxidized every tested aldehyde to some extent. The highest activities were obtained with phenylacetaldehyde and benzaldehyde, but also many aliphatic aldehydes like acetaldehyde, propionaldehyde, crotonaldehyde, and glyceraldehyde were turned over with high rates. The rates were somewhat lower for substituted aromatic aldehydes, glutardialdehyde and formaldehyde (Table 3).

**Table 3:** Substrate spectrum of AOR*_Aa_*.

Substrate	Spec. activity	Electron
Substrate	(μmol min^-1^ mg^-1^)	acceptor
Phenylacetaldehyde	25.1	BV
Benzaldehyde	23.6	BV
Acetaldehyde	19.9	BV
Propionaldehyde	12.3	BV
Crotonaldehyde	12.0	BV
Glyceraldehyde	10.3	BV
2-Aminobenzaldehyde	6.58	BV
Glutardialdehyde	6.11	BV
Formaldehyde	4.14	BV
*p*-Hydroxybenzaldehyde	3.74	BV
Benzaldehyde	12.0	NAD^+^
Phenylacetaldehyde	11.2	NAD^+^
Acetaldehyde	9.60	NAD^+^
Crotonaldehyde	8.95	NAD^+^

*Substrate specificity of AOR from A. aromaticum EbN1 with either benzyl viologen (BV) or NAD^+^ as electron acceptors. Standard deviations were less than 15%.*

Finally, we determined the kinetic parameters for the oxidation of three model substrates with either BV or NAD^+^ as electron acceptors. The selected substrates consisted of one aliphatic and two aromatic aldehydes, acetaldehyde, phenylacetaldehyde, and benzaldehyde. All of them exhibited a 2 to 2.6-fold higher apparent maximal turnover rate with BV than with NAD^+^ as electron acceptor and their kinetics could be fitted well against the Michaelis–Menten equation (Table 4). The measured *V*_max_ values were similar to the previously observed rates in the survey of various substrates (Table 4) and represent apparent turnover numbers (*k*_cat_ values) of 81–103 s^-1^ with BV, and of 35–43 s^-1^ with NAD^+^ as electron acceptors. The highest apparent *K*_m_ value were recorded for acetaldehyde, whereas that for phenylacetaldehyde was about 50% lower, and the lowest value was obtained for benzaldehyde. The latter substrate also showed significantly different apparent *K*_m_ values in its reactions with the two electron acceptors (sevenfold lower with NAD^+^), whereas the apparent *K*_m_ values of the other two substrates were similar with either electron acceptor. Because of the low apparent *K*_m_ values, the catalytic efficiency was best for benzaldehyde, followed by that for phenylacetaldehyde and acetaldehyde. This may be taken as indication that AOR*_Aa_* may have evolved to convert mainly aromatic aldehydes generated from the very extensive metabolism of aromatic compounds in *A. aromaticum* EbN1 (Rabus et al., 2005; Wöhlbrand et al., 2007; Rabus et al., 2014). The apparent kinetic parameters were also determined for the electron acceptors BV and NAD^+^ with phenylacetaldehyde as substrate, establishing plausible apparent *K*_m_ values of 12.5 μM for NAD^+^ and 21.2 μM for BV.

**Table 4:** Apparent kinetic parameters of AOR*_Aa_*.

Substrate	*V*_max_ [μmol min^-1^ mg^-1^]	*K*_m_ [μM]	*k*_cat_ [s^-1^]	*k*_cat_/*K*_m_ [μM^-1^ s^-1^]	*R*^2^	Electron acceptor
Phenylacetaldehyde	9.3	75	40	0.53	0.98	NAD^+^
Phenylacetaldehyde	18.9	82	81	0.99	0.96	BV
Benzaldehyde	8.0	10	35	3.50	0.86	NAD^+^
Benzaldehyde	21.4	67	92	1.38	0.94	BV
Acetaldehyde	10.1	155	43	0.27	0.83	NAD^+^
Acetaldehyde	23.8	141	103	0.73	0.89	BV
NAD^+^	12.5	63	54	0.86	0.94	n.a.
BV	21.2	23	91	3.96	0.84	n.a.

*The oxidation of three different aldehydes was assayed with either BV or NAD^+^ as electron acceptors, the apparent kinetic parameters for BV and NAD^+^ were assayed with 1 mM benzaldehyde as substrate. Values for k_*cat*_ are given per AOR holoenzyme (α_*2*_β_*2*_γ_*2*_-complex of 260 kDa). R^*2*^ values indicate the respective quality of mathematical fitting against the Michaelis–Menten equation. n.a., not applicable.*

**FIGURE 6:**
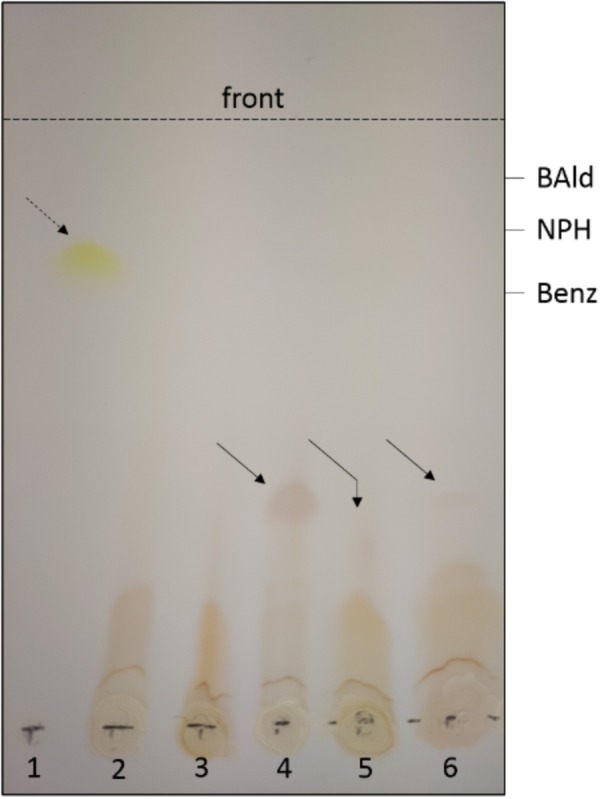
Reverse reaction of AOR*_Aa_*. Only the visible spots observed after TLC analysis are shown; the migration positions of benzoate (Benz), benzaldehyde (BAld) and 3-nitrophenylhydrazine (NPH) were detected by fluorescence quenching using the respective pure compounds as controls. The broken arrow shows the product formed from BAld and NPH in the absence, the solid arrows the product formed in the presence of Ti(III) citrate. Lanes: (1) apparent hydrazone formed from mixing BAld and NPH (without reductant); all other lanes show experiments containing 1.2 mM Ti(III) citrate; (2) control of NPH; (3) control of mixed NPH and Benz (60 mM); (4) control of mixed NPH and BAld; (5) purified AOR with NPH and Benz (100 mM), and (6) with NPH and Benz (60 mM). The slightly deviant migration of the product in lane (5) appears to be an experimental artifact, since the same sample comigrated with the control in a subsequent TLC analysis.

The previously described thermophilic AOR orthologs from Archaea or *M. thermoacetica* have been reported to slowly reduce organic acids directly to the corresponding aldehydes, if the thermodynamic equilibrium is favorable for this reaction by low pH values and/or the presence of semicarbazide or alcohol dehydrogenases removing the aldehydes from equilibrium (White et al., 1989; Heider et al., 1995; Huber et al., 1995). Therefore, we tested AOR*_Aa_* for this activity, using benzoate as substrate in the presence of 3-nitrophenylhydrazine to trap the generated aldehydes, and either reduced methyl viologen (MV) or Ti(III) citrate as electron donors. None of the experiments with dithionite-reduced MV showed any indications of generating the expected benzaldehyde (data not shown), but we identified a red colored conversion product from benzoate in assays with Ti(III) citrate by TLC analysis and compared it to products of non-enzymatic controls, in which benzaldehyde and 3-nitrophenylhydrazine had been co-incubated under the same conditions (Figure 6). All compounds present in the assay were identified by comigrating controls and showed the following *R*_f_ values: benzoate, 0.65; benzaldehyde, 0.88; 3-nitrophenylhydrazine, 0.76. Mixtures of benzaldehyde and 3-nitrophenylhydrazine yielded two different products in the absence or presence of Ti(III) citrate, which migrated with *R*_f_ values of 0.84 and 0.38, respectively (Figure 6). The product observed in the enzyme assays comigrated with the latter compound (*R*_f_ = 0.38). The apparent generation of benzaldehyde was also observed when small concentrations of MV (1–10 μM) were added to the assay [containing 1.2 mM Ti(III) citrate], but not in the presence of equimolar concentrations of both redox mediators (both 1 mM). Because of the high concentration of Ti(III) citrate and the long incubation periods necessary to detect products (2–3 h), which precluded any spectrophotometric assay, we could only estimate the rates of this reaction by comparing the intensities of the observed spots on TLC plates with various concentrations of control assays with benzaldehyde. Based on these data, we observed very slow rates of 1–20 nmol min^-1^ mg protein^-1^ for the reverse reaction of AOR*_Aa_*, which equal to less than 0.1% of the rate of the forward reaction.

## Discussion

**FIGURE 7:**
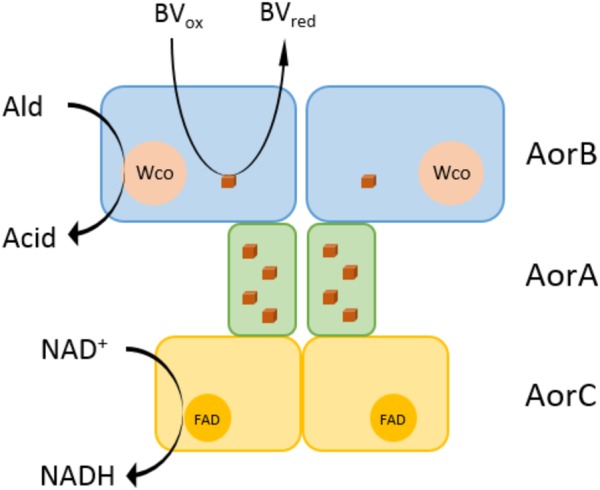
Hypothetical structure of the AOR*_Aa_* enzyme complex. AOR*_Aa_* is proposed to consist of three subunits in an α_2_β_2_γ_2_ composition. AorB is predicted to contain the tungsten cofactor (Wco) as catalytic center and one Fe_4_S_4_ cluster. The electron transfer subunit AorA contains four Fe_4_S_4_ clusters, and AorC contains an FAD cofactor, which are involved in proposed electron transfer pathways from aldehyde oxidation either to benzyl viologen or NAD^+^ as electron acceptors.

AOR*_Aa_* is the first aldehyde-oxidizing tungstoenzyme isolated from a mesophilic and facultatively anaerobic bacterium. The previously known members of this enzyme family are all from obligatory anaerobic Archaea or Bacteria, and the best characterized examples are from hyperthermophilic *Thermococcus* or *Pyrococcus* species or from the thermophilic bacterium *Moorella thermoacetica* (Huber et al., 1995; Kletzin and Adams, 1996). All of the previously characterized isoenzymes of the AOR subfamily have been characterized as homodimers of one subunit, except for AOR of *M. thermoacetica* (AOR*_Mt_*) which has been described with different subunit compositions in different studies (White et al., 1989; Strobl et al., 1992; Huber et al., 1995). Since the operon structures are identical between *A. aromaticum* EbN1 and *M. thermoacetica* it is very likely that the enzymes of both species consist of three subunits in an α_2_β_2_γ_2_ composition, as indicated in the latest available study on AOR*_Mt_* (Huber et al., 1995). We assume that the work on AOR*_Mt_* suffered from the same complications we experienced with AOR*_Aa_*, which was only accessible in sufficient amounts for proper purification after the cells were forced to produce it for Phe degradation after deleting the *pdh* gene (Schmitt et al., 2017). Both AOR*_Aa_* and AOR*_Mt_* do not contain two typical conserved amino acids of archaeal AOR which are involved in binding a single bridging iron ion between the subunits of the homodimer, providing additional evidence for a structural difference. The composition of AOR*_Aa_* as heterohexamer of three different subunits led us to propose a hypothetical structure of the enzyme as shown in Figure 7, which connects the active site W-cofactor in AorB via the Fe-S clusters of AorB and AorA to the FAD cofactor in AorC, which represents the most likely reactive site for electron transfer to NAD^+^. The alternative electron acceptor BV probably receives electrons by interacting directly with one of the Fe-S clusters of the electron transfer pathway, as assumed for the archaeal AORs (Kletzin and Adams, 1996).

AOR*_Aa_* shows the typical broad substrate specificity recognized for archaeal AOR or AOR*_Mt_* (Heider et al., 1995; Huber et al., 1995). We have already shown previously that *A. aromaticum* EbN1 produces this enzyme under various growth conditions, especially when a substrate is degraded via an aldehyde intermediate, for example during degradation of benzyl alcohol or benzaldehyde (Schmitt et al., 2017). These observations substantiate a potential primary function of AOR-type enzymes in aldehyde detoxification (Kletzin and Adams, 1996; Schmitt et al., 2017). The observed reactivity of AOR*_Aa_* with BV as electron acceptor indicates that a ferredoxin acts as natural electron acceptor in the cells, as known for archaeal AOR (Kletzin and Adams, 1996). Since the genome of *A. aromaticum* EbN1 contains multiple genes for ferredoxins (Rabus et al., 2005), we still have to identify which ferredoxin may be physiologically linked to AOR*_Aa_*. This will then open up further studies to study the reaction for either competitive or synergistic effects of the two electron acceptors, ferredoxin and NAD^+^. The observed differences in specific activities with either BV or NAD^+^ as electron acceptor may either reflect different preferences of AOR*_Aa_* for the respective reactants or may be a consequence of the recorded partial lack of FAD cofactors, which may slow down the reaction with NAD^+^.

A very profound difference between AOR*_Aa_* and archaeal AORs is the observed high tolerance against oxygen. While AOR isoenzymes from Archaea belong to the most oxygen-sensitive enzymes known, the enzyme complex from *A. aromaticum* EbN1 retained full activity after 24 h of exposure to air in extracts and was inactivated only relatively slowly as a purified enzyme, using BV as electron acceptor. Since a similar half-life time after exposure to air as observed for AOR*_Aa_* was also reported for AOR*_Mt_* (Huber et al., 1995), it may be speculated that the additional redox cofactors in these enzymes complex could be involved in protecting the active site from damage by reactive oxygen species by keeping it in the oxidized state. The additional stabilization in cell extract that we observed for AOR*_Aa_* is obviously mediated by other factors, probably by consuming oxygen fast enough to prevent it from interacting with AOR*_Aa_*.

Another obvious difference of AOR*_Aa_* to archaeal AOR relates to the temperature dependence of the enzymes. Looking at similar predicted enzymes form sequenced genomes, AOR*_Aa_* belongs to a large branch of enzymes from mesophilic bacteria with an aerobic or denitrifying physiology. The data on AOR*_Aa_* showed a temperature optimum of 40°C, which is 10°C higher than the maximum growth temperature of *A. aromaticum* EbN1 (Rabus and Widdel, 1995), whereas the enzymes form hyperthermophilic archaea are virtually inactive at these temperatures and reach their optima only at 90–100°C (Mukund and Adams, 1991; Heider et al., 1995). Interestingly, the structurally related AOR*_Mt_* shows a very similar temperature optimum of 46°C (Huber et al., 1995), despite coming from a thermophilic bacterium with an optimum growth temperature of 60°C (Wiegel, 2009). Adaptation of AOR*_Mt_* to higher temperatures seems to consist in a shallower temperature-dependent deactivation curve than observed for AOR*_Aa_*, because the enzyme was reported to show still 60% activity at 70°C (Huber et al., 1995). Therefore, the complex “bacterial” type of AOR may be better suited for reactivity at lower to moderately thermophilic temperatures, whereas the more compact homodimeric structures of archaeal AOR may confer more intrinsic stability against denaturation, allowing reactivity at higher temperatures.

A characteristic property only known from members of the subfamily of AOR-like enzymes is their ability to catalyze the reverse reaction, direct reduction of acids to the respective aldehydes (Kletzin and Adams, 1996; Basen et al., 2014). In particular, this has been observed for archaeal AOR, using reduced MV (Heider et al., 1995), and for AOR*_Mt_*, using reduced carbamoyl methyl viologen as electron donor, both at low pH values around pH 5–6 (Huber et al., 1995). However, the rates of the reverse reactions were very low and ranged only at less than 0.3% of the respective aldehyde oxidation rates. We did not observe acid reduction by AOR*_Aa_* under the previously described conditions, but needed to set up even more favorable reaction conditions, including low pH, high substrate concentrations, trapping of the aldehydes by hydrazine derivatives, and applying the strong reductant Ti(III) citrate (E^∘^′ = -480 mV) to observe very slow reduction of benzoate to benzaldehyde. Therefore, the discrepancy between the rates of aldehyde oxidation and the estimated value of acid reduction seems to be even larger for AOR*_Aa_* than for the previously investigated AORs. A potential explanation of these differences might involve the influence of temperature on the thermodynamics of the process. Using Gibbs enthalpies of formation Δ*G*_f_^∘^′ (Dean, 1992), a redox potential of *E*^∘^′ = -0.51 V can be calculated for the redox couple benzaldehyde/benzoic acid (Δ*G*_f_^∘^′ is only available for undissociated benzoic acid). This calculated value is a good estimation for the upper limit for the couple benzaldehyde/aqueous benzoate, since the experiments were performed at pH values close to its pK_a_ (4.2). For comparison, the calculated standard potential of the redox couple acetaldehyde/acetic acid is *E*^∘^′ = -0.51 V, whereas those for the couple acetaldehyde/acetate are *E*^∘^′ = -0.62 V at pH 7 and *E* = -0.56 V at pH 5 (using Δ*G*_f_^∘^′ values from Thauer et al., 1977). Therefore, benzoate (or acetate) reduction is endergonic under standard conditions with either reduced MV (*E*^∘^′ = -0.44 V) or Ti(III) citrate (*E*^∘^′ = -0.48 V) as electron donor and only becomes feasible under non-standard conditions according to Δ*G*′ = Δ*G*^∘^′ + RT lnK. To achieve this, the equilibrium term *K* needs to yield a negative lnK value (i.e., higher substrate concentrations than those of the products to pull the reaction), and higher temperatures promote this effect. Therefore, the stronger reductant Ti(III) citrate may be required to catalyze acid reduction at room temperature, while reduced MV is sufficient under hyperthermophilic conditions.

The potential direct reduction of acids to aldehydes by AOR-like enzymes has recently been recognized as interesting biotechnological asset, because this would enable the production of alcohols from syngas, a cheap resource consisting of CO, CO_2_, and H_2_. Acetogenic microorganisms are able to grow by converting syngas to acetate, but do not generate enough energy to allow an ATP-dependent activation of acetate, which is required for any other known pathway of acid reduction. It has already been shown that fermentation pathways normally ending with acetate can indeed be redirected toward ethanol generation by coupling an endogenous acetate-reducing AOR reaction to the further reduction of acetaldehyde to ethanol by a recombinantly synthesized alcohol dehydrogenase in *Pyrococcus furiosus* (Basen et al., 2014; Keller et al., 2017). AOR*_Aa_* and the related enzymes from mesophilic bacteria may be employed in similar processes in mesophilic oxygen-tolerant organisms, which should be handled with much more ease in biotechnology laboratories than strictly anaerobic hyperthermophiles.

A view on the amino acid sequence relatedness of the large subunits of the members of the AOR enzyme family (Figure 1) shows that archaeal and bacterial AOR should be regarded as separate subfamilies in addition to the previously known ones, represented by FOR, GAPOR, WOR4, WOR5, XOR, and benzoyl-CoA reductase (BamB subunit). All these subfamilies are clearly distinct entities, as indicated by the respective sequence identity values, which are shown in Table 5 for the subunits of AOR*_Aa_* and some selected members of the various subfamilies. Note that the previously known archaeal AORs also contain some sequences from anaerobic bacterial species, such as *E. acidaminophilum*, while the bacterial AORs are found in aerobic/denitrifying mesophilic bacteria. As judged from the organization of the respective operons, all enzymes of the archaeal AOR branch are homodimeric, whereas all bacterial AORs are composed of the three subunits found in AOR*_Aa_*. Curiously, AOR*_Mt_*, which represents the only characterized example of a three-subunit AOR from a strictly anaerobic and (moderately) thermophilic bacterium, seems to be a special case, branching out between the two AOR subfamilies (Figure 1). This feature may be connected with AOR*_Mt_* evolving toward a more temperature-resistant variant and adopting some of the sequence modules of the archaeal AORs. In addition to the bacterial AOR subfamily (plus AOR*_Mt_*), only the benzoyl-CoA reductases and the XOR subfamily appear to harbor additional subunits. The benzoyl-CoA reductases are known to form huge complexes in which the W-containing subunit is associated as a sub-module with a small Fe-S cluster containing subunit (Weinert et al., 2015), and the XOR-like enzymes also appear to contain an additional small subunit containing Fe-S clusters (Scott et al., 2015). However, the respective sequences of these additional subunits do not show large similarities to the small subunits of bacterial AORs (Table 5), suggesting that the acquisition of the extra subunits may have occurred independently in the different subfamilies.

**Table 5:** Identities of the subunits of selected members of the AOR family in relation to those of AOR*_Aa_*.

Species	Type	AorA	AorB	AorC
*Aromatoleum aromaticum* EbN1	AOR	100%	100%	100%
*Acidovorax* sp. JS42	AOR	69%	74%	62%
*Moorella thermoacetica*	AOR	40%	54%	34%
*Pyrococcus furiosus*	AOR	n.a.	50%	n.a.
	FOR	n.a.	34%	n.a.
	WOR4	n.a.	34%	n.a.
	WOR5	n.a.	31%	n.a.
	GAPOR	n.a.	20%	n.a.
*Thermococcus paralvinellae*	AOR	n.a.	50%	n.a.
*Caldicellulosiruptor bescii*	XOR	29%	27%	n.a.
*Geobacter metallireducens*	BamBC	27% (BamC)	29% (BamB)	n.a.

*The values indicate sequence identities between the corresponding subunits between AOR_*Aa*_ and members from the various AOR subfamilies (see Figure 1). Potential additional subunits homologous to AorA or AorC are included when they are encoded in a common operon with the gene for the respective tungsten-containing subunit. n.a., not applicable.*

## Author Contributions

JH, FA, and GS designed the research project and interpreted the data. FA and GS performed most experiments. AW and MS contributed to AOR activity tests. AS performed the ICP-MS. JK performed the MALDI-TOF analysis. JH and FA wrote the manuscript.

## Conflict of Interest Statement

The authors declare that the research was conducted in the absence of any commercial or financial relationships that could be construed as a potential conflict of interest.

## Footnotes

1 https://www.ebi.ac.uk/Tools/msa/clustalo/

2 https://itol.embl.de/
