# Supplementary material for: Characterization of an Aldehyde Oxidoreductase From the Mesophilic Bacterium Aromatoleum aromaticum EbN1, a Member of a New Subfamily of Tungsten-Containing Enzymes
Source: Front Microbiol. 2019 Jan 31;10:71. doi: 10.3389/fmicb.2019.00071 (PMC6365974; doi:10.3389/fmicb.2019.00071)
Supplement: Supplementary file 1 [file Data_Sheet_1.PDF]

Supplementary Material

Characterisation of an aldehyde oxidoreductase from the mesophilic bacterium *Aromatoleum aromaticum* EbN1, a member of a new subfamily of tungsten-containing enzymes

Fabian Arndt[1], Georg Schmitt[1], Agnieszka Winiarska[2], Martin Saft[1], Andreas Seubert[3], Jörg Kahnt[4], Johann Heider[1,5#]

[1] Philipps-Universität Marburg, Faculty of Biology, Karl-von-Frisch Straße 8, D-35043 Marburg, Germany

[2] Jerzy Haber Institute of Catalysis and Surface Chemistry, Polish Academy of Sciences, Niezapominajek 8, PL-30239 Krakow, Poland

[3] Philipps-Universität Marburg, Faculty of Chemistry, Hans-Meerwein-Str. 4, D-35043 Marburg, Germany

[4] Max-Planck-Institut für terrestrische Mikrobiologie, Karl-von-Frisch Straße 10, D-35043 Marburg, Germany

[5] LOEWE Center for Synthetic Microbiology, Philipps-Universität, D-35043 Marburg, Germany

[#] Author for correspondence

e-mail: heider@biologie.uni-marburg.de

Table S1: Accession numbers for phylogenetic analysis of the AOR family. The subfamily affiliations and source organisms of the selected entries are indicated.

Subfamily	Species	Accession number
**Bacterial AOR**	*Aromatoleum aromaticum* EbN1	WP_011238652
**Bacterial AOR**	*Acidovorax sp. JS42*	WP_011807029
**Bacterial AOR**	*Azoarcus toluclasticus*	WP_018990435
**Bacterial AOR**	*Caldimonas manganoxidans*	WP_019559976
**Bacterial AOR**	*Methyloversatilis discipulorum*	WP_020164287
**Bacterial AOR**	*Methyloversatilis universalis*	WP_018229612
**Bacterial AOR**	Candidatus *Accumulibacter sp.* SK-02	KFB75060
**Bacterial AOR**	*Acidovorax sp.* JS42	WP_011807029
	Moorella thermoacetica	WP_071520516
**Archaeal AOR**	*Pyrococcus furiosus*	WP_011011461
**Archaeal AOR**	*Thermococcus paralvinellae*	WP_042681437
**Archaeal AOR**	*Hippea jasoniae*	WP_035589419
**Archaeal AOR**	*Carboxydothermus hydrogenoformans*	WP_011343719
**Archaeal AOR**	*Eubacterium acidaminophilum* (*Peptoclostridium acidaminophilum*)	WP_025436835
	Pyrobaculum aerophilum	AAL62901
	Desulfovibrio gigas	WP_021759675
	Clostridium formicoaceticum	WP_070964744
**XOR**	*Caldicellulosiruptor bescii*	WP_013403731
**XOR**	*Methanospirillum hungatei*	WP_011448034
**XOR**	*Coprothermobacter platensis*	WP_018963763
**GAPOR**	*Pyrococcus furiosus*	WP_011011581
**GAPOR**	*Pyrococcus abyssi*	WP_010868698
**GAPOR**	*Thermococcus onnurineus*	WP_012572460
**BamB**	*Geobacter metallireducens*	WP_004514579
**BamB**	*Geobacter metallireducens*	WP_004512044
**FOR**	*Pyrococcus furiosus*	WP_014835355
**FOR**	*Pyrococcus_abyssi*	WP_010868317
**FOR**	*Thermofilum sp.* ex4484 82	OYT29225
**WOR4**	*Pyrococcus furiosus*	WP_011013103
**WOR4**	*Thermococcus celericrescens*	WP_058938613
**WOR4**	*Palaeococcus ferrophilus*	WP_048150761
**WOR5**	*Pyrococcus furiosus*	WP_014835423
**WOR5**	*Thermococcus litoralis*	WP_004070105
**WOR5**	*Thermococcus sibiricus*	WP_048160352

Table S2: MALDI-TOF analysis of AOR$_{Aa}$ subunits. Peptide count and protein score values of the three subunits after separation by SDS-PAGE are given with corresponding accession numbers. The identity of proteins with AorABC is evident from their high protein scores, while potential co-migrating contaminants are represented by much lower protein score values.

Spot	Name	Accession number	Peptide count	Protein score
**1**	AorB	WP_011238652	39	1030
**2**	AorC	WP_018990436	29	877
	phenylglyoxylate:acceptor oxidoreductase	WP_041646480	24	110
**3**	AorA	WP_011238651	12	289
	SecB	WP_011236410	5	70
